# PReS-FINAL-2308: Catatonia due to systemic lupus erythematosus: characteristics and 36 months follow up of this rare manifestation of disease

**DOI:** 10.1186/1546-0096-11-S2-P298

**Published:** 2013-12-05

**Authors:** V Selmanovic (Mulaosmanovic), T Avčin, S Mesihović-Dinarević, A Omerčahić-Dizdarević, L Oruč, S Zubčević, D Miličić-Pokrajac, D Anić, A Mornjaković-Franca, S Tanović, A Bučan-Varatanović, A Čengić

**Affiliations:** 1 Allergology, Rheumatology and Clinical Immunology, Children's Hospital Sarajevo, Sarajevo, Bosnia and Herzegovina, Sarajevo, Bosnia and Herzegovina; 2 Allergology, Rheumatology and Clinical Immunology, Children's Hospital Ljubljana, Ljubljana, Slovenia, Sarajevo, Bosnia and Herzegovina; 3 Psychyatry, Department of Psychiatry, University Clinical Center, Sarajevo, Bosnia and Herzegovina; 4 Neuropediatric, Children's Hospital Sarajevo, Sarajevo, Bosnia and Herzegovina; 5 Nephrology, Children's Hospital Sarajevo, Sarajevo, Bosnia and Herzegovina; 6 Pediatric Intensive Care Unit, Children's Hospital Sarajevo, Sarajevo, Bosnia and Herzegovina; 7 Pediatric Radiology, Institute of Radiology, Sarajevo, Bosnia and Herzegovina; 8 Neurology, Children's Hospital Sarajevo, Sarajevo, Bosnia and Herzegovina; 9 Child Psychology, Children's Hospital Sarajevo, Sarajevo, Bosnia and Herzegovina

## Introduction

Catatonia is a rarely reviewed clinical feature of neuropsychiatric (NP) manifestation of pediatric systemic lupus erythematosus (pSLE). It is a state of neurogenic motor immobility, and behavioral abnormality manifested by stupor.

## Objectives

Our goal is to present catatonia as rare NP manifestation of pSLE; to report success of immunosupresive therapy, to underline ultimate need for multidisciplinary team approach.

## Methods

We describe a 15,5 y old girl presented with fever and abdominal pain in June 2009. Patient had numerous scleronodous skin lesions, developed 20 months ago, treated as localized scleroderma in another center. She rapidly developed malar rash, periungval erythema, extreme conjunctival injection, photophobia, soft palate erosions, pericardial effusion, mild vaginal bleeding, intraarticular effusion, became excitable, moody, malaise, accompanied with positive immunoserology. Signs of incomplete macrophage activation syndrome were present (like feritin 162 098, exc.). Diagnosed as SLE, peroral steroids started. Afebrile in next 24 hours, cheerful, with good general condition. On therapy day 13., dramatic qualitative change of conscious level with psychomotor disturbance (resembling extrapyramidal symptomatology), fear, visual hallucinations, followed by tachycardia and hypertension. Organic catatonia and mutism developed. Brain CT, MRI, MRA were normal. Received pulses of metil-prednisolone and cyclophosphamide, IVIG, hydroxiquinolon-sulphat, aspirin, bensodiasepins, supportive therapy.

## Results

Patient had excellent therapy response. Catatonia took 4 months for complete recovery. Lost 8 kg of body weight in 7 days, several months of sinus tachycardia were consistent with CNS-lupus. Several weeks prolonged hypertension was due to Lupus nephritis class III (confirmed 5 months-biopsy). Retrospective medical records analisis showed skin biopsy performed in jan 2008 was consistent with LE profundus as well as positive immunoserology on few occasions positive ANA, anti-ds-DNA, antiphospholipid antibodies.

## Conclusion

Catatonia is one of multitude of NP syndromes reported in SLE patients. The mechanisms are related to auto-antibody-mediated neurotoxicity. 90% of patients who developed psychotic symptoms had cutaneous involvement. Positive antiphospholipid antibodies are strongly related with NP manifestation.

## Disclosure of interest

None declared.

